# Radiofrequency ablation of osseous metastases for the palliation of pain

**DOI:** 10.1007/s00256-007-0404-5

**Published:** 2008-03-01

**Authors:** L. Thanos, S. Mylona, P. Galani, D. Tzavoulis, V. Kalioras, S. Tanteles, M. Pomoni

**Affiliations:** Department of Interventional Radiology—CT, Hellenic Red Cross Hospital, 1, Athanassaki Street, 115 26 Athens, Greece

**Keywords:** Radiofrequency ablation, Osseous metastases, Minimally invasive treatment, Pain

## Abstract

A number of different methods have been proposed for pain relief in cancer patients with bone metastases, each with different indications, contraindications and complications (systemic analgesics, bisphosphonates, antitumor chemotherapy, radiotherapy, systemic radio-isotopes, local surgery and vertebroplasty). The ideal treatment has to be fast, safe, effective and tolerable for the patient. CT-guided radiofrequency (RF) ablation may fulfill these criteria. Our experience in the treatment of 30 patients (34 lesions) with painful bone metastases using RF ablation was assessed. There was a significant decrease in the mean past-24-h Brief Pain Inventory (BPI) score for worst pain, for average pain and for pain interference during daily life (4.7, 4.8 and 5.3 units respectively) 4 and 8 weeks after treatment. There was a marked decrease (3 out of 30 patients 4 and 8 weeks after treatment) in the use of analgesics. CT-guided RF ablation appears to be effective for treatment of painful bone metastases.

## Introduction

Painful bone metastases are a common cause of morbidity in patients with metastatic cancer, especially when combined with possible neural compression and pathologic fractures. Several solid cancers are associated with bone involvement, most often, prostate and breast. Thirty to seventy percent of cancer patients develop bone metastases [1]. They indicate widespread disease. Treatment of local disease may reduce the pain of these patients who, in most cases, have a life expectancy of months. Such treatment must be fast, safe, effective and tolerable.

A number of treatment methods are available that have variable success and complications. Radiation therapy is the preferred treatment in this setting, but other modalities such as chemotherapy, hormonal therapy, radiopharmaceutical therapy and surgery—alone or in combination with non-steroid anti-inflammatory drugs (NSAIDs), opioids and adjuvant drugs—are used for pain palliation [1–3].

Radiofrequency (RF) ablation is a relatively new method for the treatment of painful bone metastases. Previously, tumour ablation was performed with percutaneous ethanol injection under CT guidance [4]. Administration of 95% ethanol was described in 25 terminally ill cancer patients with 27 bone lesions who had been unsuccessfully treated by radiation therapy and/or chemotherapy.

Radiofrequency ablation has been employed for the treatment of hepatocellular carcinoma (HCC), liver metastases, renal and lung tumours, as well as for the treatment of osteoid osteoma, for which it has become the treatment of choice [1–3]. Competing methods include chemical ablation (with ethanol or acetic acid) and thermal therapies, such as with laser, microwave, ultrasound and cryoablation [5]. The aim of this study was to demonstrate the effectiveness of RF ablation of bone metastases using CT guidance.

## Materials and methods

Thirty patients were retrospectively identified. There were 19 men and 11 women. Their ages were between 47 and 91 years (mean ± standard deviation [SD]: 66.53 ± 10.56 years). The patients had bone metastases, which were treated with RF ablation under CT guidance, at our hospital, over a period of 4 years. All treated lesions were osteolytic with a combination of bone destruction and a soft tissue mass. In 26 there was a solitary lesion, and in 4 patients there were two such lesions, resulting in a total number of 34 metastases. Bone metastases were diagnosed by bone scintigraphy and spiral CT. The diagnosis was confirmed with a core biopsy obtained at the beginning of the procedure. Their topographical distribution and the originating primary malignancies are presented in Table 1. In our study the most common treated metastases originated from colon cancer, which was probably related to the patient population treated at the oncology department of our hospital.

**Table 1:** Bone metastasis classification, with regard to the primary malignant lesion and the site of the skeleton involved

Site of primary neoplasm origin	*n*	Site of metastasis	*n*
Colon	13	Pelvis	15
Breast	7	Ribs	6
Prostate	2	Sacrum	5
Lung	4	Femur	3
Renal	2	Spine	3
Thyroid	1	Scapula	2
Skin melanoma	1	Tibia	1
Total	30	Total	34

Lesion diameter was between 1 and 14 cm (mean ± SD: 3.9 ± 2.6 cm). For sizes over 3 cm, two or more electrode placements were needed (with a maximum of five). Previously obtained imaging examinations were evaluated for lesion characteristics and feasibility of electrode positioning and ablation. Lesions located in proximity to the spinal cord and major nerves (less than 1 cm) were excluded from RF treatment. Patient selection criteria are summarised in Table 2. The study was in accordance with the ethical principles of the Helsinki Declaration and informed consent was obtained in each case.

**Table 2:** Criteria for the selection of patients to undergo radiofrequency ablation

Patient selection criteria
Brief Pain Inventory (BPI) score above 4
Lesions not responding to chemotherapy and/or radiation therapy (completion of therapy at least 3 weeks before the radiofrequency ablation session)
Chemotherapy-associated complications that halted this treatment
Lesions adjacent to structures sensitive to irradiation
Patients with life expectancy greater than 2 months who were not eligible for surgical treatment
Patients who preferred this treatment over the other alternatives

Physical examination was performed by the oncologist and in collaboration with the radiologist performing the ablation. Pain was assessed with the Brief Pain Inventory (BPI) The use of analgesics was recorded the day before the procedure.

Before the procedure blood cell count and blood clotting analysis were performed. Minimal requirements were: platelet (PLT) count >50,000/ml (normal range, 150,000–350,000/ml); prothrombin time (PT), international normalised ratio (INR) <1.3 (normal range, 0.8–1.2); and partial thromboplastin time (PTT) <34 s (normal range, 25–34 s). The procedure was performed under conscious sedation (administration of 3 mg of bromazepam PO and 50 mg of pethidine hydrochloric acid intramurally, 45 min prior to the procedure) and was trained in regular breathing and breath-holding (suspended respiration) before the procedure. He/she was placed in the appropriate position (prone, supine, or lateral, depending on the site of the lesion) and a scan of the desired area with a 5-mm slice thickness was performed, using a Picker 5000® (Philips Medical Systems, Amsterdam, The Netherlands).

At least one of two staff radiologists with extensive experience in biopsies and tumour ablations was involved in all ablations.

The lesion’s exact location and depth, in relation to the overlying skin, was determined on CT. The skin was then prepared with povidone iodine (10%) solution. Local anaesthesia (15 ml of 2% lidocaine hydrochloride solution) was administered.

Radiofrequency ablation was performed with a RITA Model 1500® electrosurgical generator (RITA Medical Systems, Mountain View, CA, USA) and a seven-array, 2- to 3-cm multitined electrode for lesions smaller than 3 cm (20 out of 34), or a nine-array multitined electrode for larger lesions (14 out of 34). The electrode tip was inserted to approximately 1 cm from the centre of the target. The electrodes were then deployed slowly, taking into account the need to ablate the lesion–bone interface. The net ablation time was ~15 min at an energy level of 90–110 W, with the target goal temperature set to 80–110°C. During the procedure the infusion port of the electrode was flushed with a 2% lidocaine hydrochloride solution in order to reduce patient discomfort and to decrease tissue overheating and vaporisation. The number of electrode placements, individual (per electrode) and total ablation times, the total energy delivered to the target and the lesion temperatures achieved were recorded.

After each session a dual-phase spiral CT examination with intravenous contrast medium was performed in order to assess response, as confirmed by low lesion attenuation values and lack of contrast enhancement.

Patients were hospitalised and observed for 24 h. Analgesics were administered if required. Before patient discharge the pain was re-evaluated with the BPI score. Post-ablation assessment was completed with telephone interview after one, four and eight week. The BPI score and the use of analgesics were recorded again.

## Results

For lesions smaller than 3 cm (20 out of 34), one placement was adequate, while for the remaining 14 out of 34 cases of lesions that were larger than 3 cm, two or more placements were required; one lesion sized 14 cm required five placements, accomplished in two sessions (since more deployments are required in larger lesions). The total procedure time ranged between 33 and 65 min (mean ± SD: 42 ± 11 min; Table 3). There were no complications and post-treatment CT revealed a good response, as confirmed by low lesion attenuation values and a lack of contrast medium enhancement, consistent with necrosis. Post-procedural CT did not demonstrate any major complications (such as haemorrhage, thrombosis of neighbouring veins, or skin burns).

**Table 3:** Lesion characteristics andtreatment

Number of lesions	Size of lesion (cm)	Number of electrode placements	Time of radiofrequency energy deposition (min)
15	<3	1	5
5	3	1	5
7	4	2	7
3	5	2	8
1	6	3	9
1	8	4	10
1	9	4	10
1	14	5	15
Total 34			

**Fig. 1:**
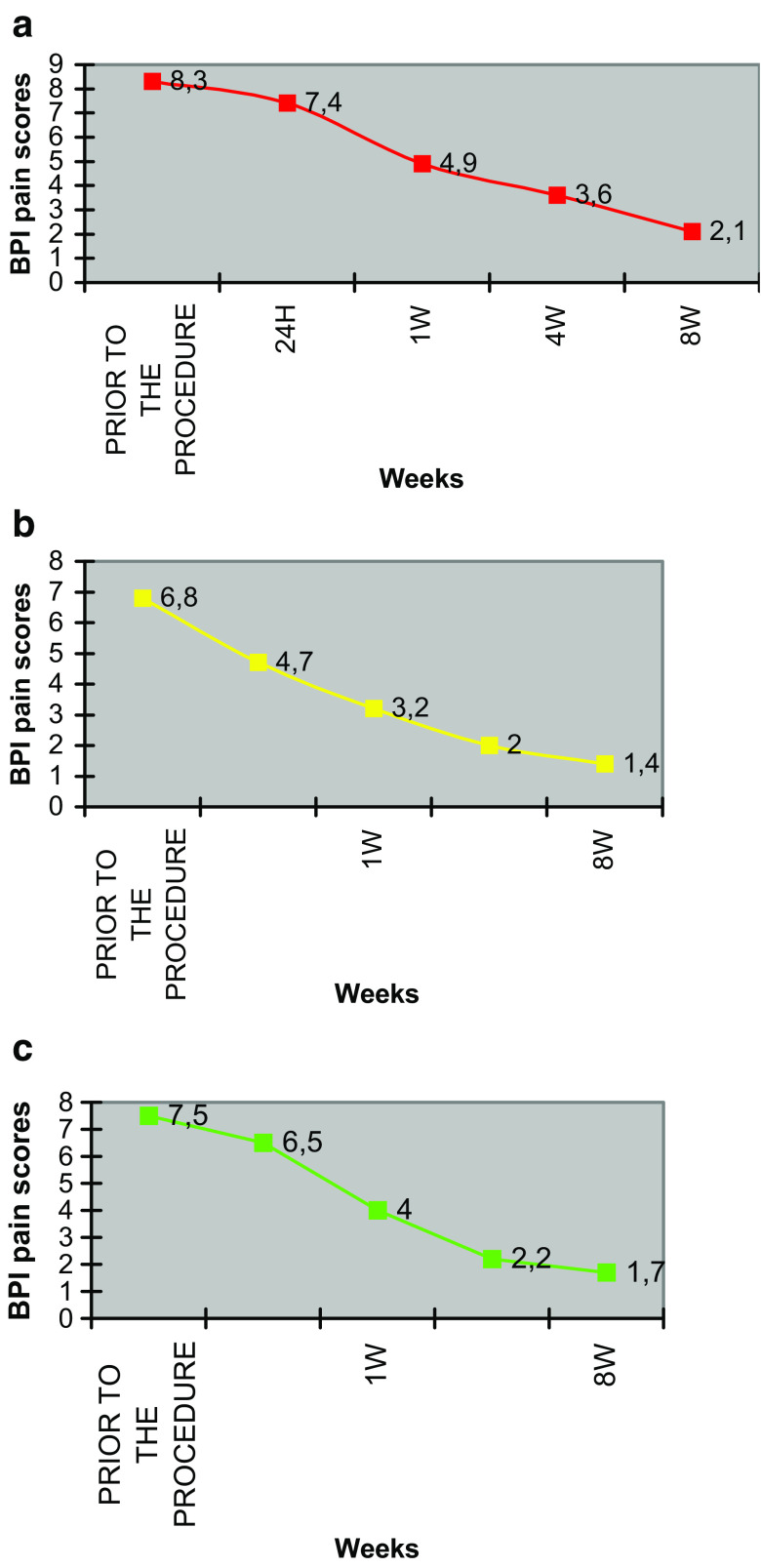
Mean Brief Pain Inventory (BPI) scores over time for patients treated with radiofrequency ablation. **a** Worst pain. **b** Average pain. **c** Interference of pain in everyday life

**Fig. 2:**
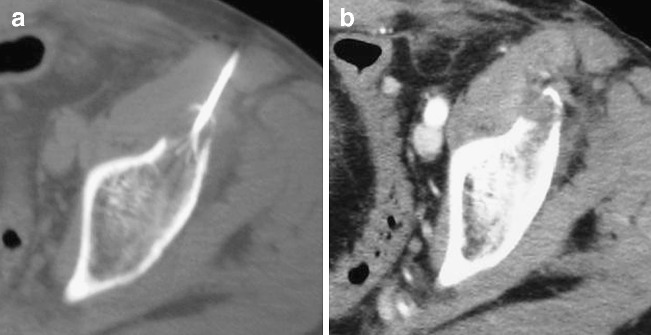
Computed tomography scan **a** during and **b** after the radiofrequency session with the patient in a supine position. The electrode is deployed inside the metastatic lesion of the left acetabulum (metastasis from breast cancer). There is no enhancement after intravenous contrast media administration. Before the radiofrequency session, the average pain score was 7. During the first 24 h after radiofrequency, it was 4 and 1, 4 and 8 weeks later the average pain scores were 3, 2 and 0 respectively

**Fig. 3:**
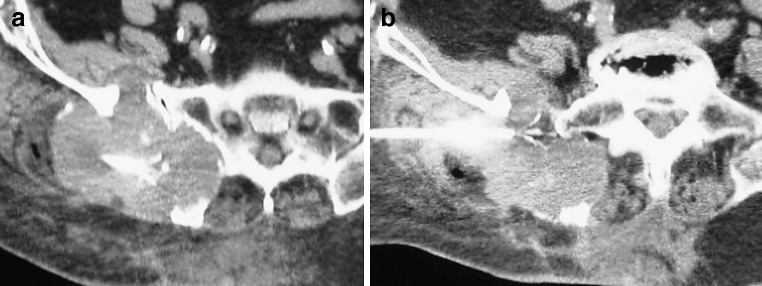
Computed tomography scan images at the level of the sacrum show **a**, **b** two different electrode placements within a soft tissue mass involving the sacrum and right iliac bone (metastasis from thyroid cancer). Before the radiofrequency session, the average pain score was 7. During the first 24 h after radiofrequency, it was 4 and 1, 4, and 8 weeks later the average pain scores were 3, 2 and 1 respectively

**Fig. 4:**
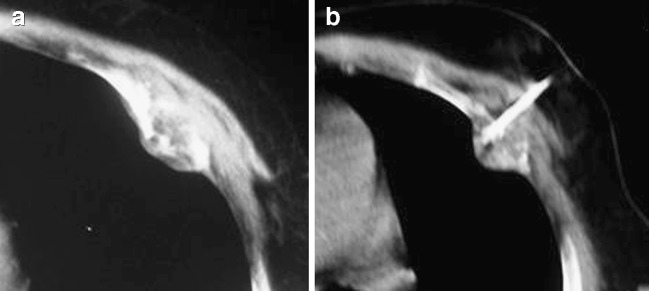
Computed tomography scan image during the electrode placement inside **a** a metastatic lesion involving a rib (the primary neoplasm originates in the lung). Immediately after the radiofrequency session the contrast enhancement CT scan revealed a hypodense area (necrosis) inside the lesion. Before the radiofrequency session, the average pain score was 6. During the first 24 h after RF, it was 4, and 1, 4 and 8 weeks later the average pain scores were 2, 2 and 1 respectively

Eleven patients reported no pain reduction during the first 24 h after the procedure and were treated with analgesics (opioids or an opioid/NSAID combination). Nineteen of the 30 patients reported early pain reduction. In none of the patients was increased pain reported. Prior to the procedure, the mean past-24-h BPI score for worst pain was 8.3 (on a numerical rating scale where 0 indicates no pain, and 10 indicates worst pain imaginable), mean pain was 6.8, and mean pain interference with daily life 7.5. These scores were reduced to 7.4, 4.7 and 6.5 24 h after the procedure, dropped to 4.9, 3.2 and 4.0 after 1 week, to 3.6, 2.00 and 2.2 after 4 weeks, and to 2.1, 1.4 and 1.7 after 8 weeks respectively. These results revealed a marked decrease in pain with subsequent improvement in the life quality for all participants since the first week post-treatment that lasted throughout the 8-week follow-up (Fig. 1). For all time points, the mean past-24-hour BPI score for worst pain, for average pain and for pain interference in daily life improved in comparison to preprocedural symptoms (*p* < 0.001, paired *t* test; Figs. 2, 3, 4).

Prior to RF ablation 27 out of 30 patients received opioids or an opioid/NSAID combination. The remaining 3 patients received NSAIDs. One week after treatment 5 out of 30 patients were treated with a combination of NSAID/low-dose opioids. Six out of thirty used NSAIDs. After 4 and 8 weeks only 3 out of 30 patients received any medication NSAIDs. One patient died during the 8-week follow-up for reasons not related to RF ablation.

## Discussion

In patients with cancer, pain originating from bone metastases can be difficult to treat. A number of treatment options are available, including NSAIDs, opioids, and adjuvant drugs medications, radiation therapy, chemotherapy, hormonal therapy, radiopharmaceutical therapy, surgery and vertebroplasty.

Medication represents the first line of treatment. NSAIDs and adjuvant drugs represent basic medication, potentially followed by NSAID/low-dose opioid combinations, and finally increasing the opioid dose.

Radiation therapy is another treatment option that may also be employed in pathologic or impending fractures [6]. Approximately 70% of patients undergoing radiation therapy will experience pain relief after between 2–3 days and up to 4 weeks after treatment. However, radiation therapy may also cause complications, mostly from damage of adjacent soft tissues [6].

Chemotherapy and radiopharmaceutical therapy are the only systemic methods of treatment that deal with even small foci of metastatic cells. However, not all metastases are sensitive to chemotherapeutic agents. Chemotherapy is often not well tolerated and is associated with complications. Radiopharmaceutical therapy can be more useful in treating patients with multifocal bone metastases. It has been reported that radiopharmaceuticals proved efficient in pain palliation mostly in bone metastases from breast, prostate and perhaps small cell lung cancer. As in the case of chemotherapy, all agents have advantages and possible side effects. Radiopharmaceutical agents vary with regard to the analgesic efficacy, duration of pain palliation, ability to repeat treatments, toxicity and expense [7].

The term “ablation” refers to the local destruction of the tumour by the means of application of either chemical agents (ethanol, acetic acid), or local deposition of some form of energy (radiofrequency, laser, microwave, ultrasound and cryoablation). Image-guided RF ablation is currently used for the treatment of various tumours with good results. According to preliminary results by Dupuy et al. [8], RF ablation can provide palliative treatment for patients with painful osseous metastases. Later on, Callstrom et al. [9] reported results after treating 12 patients and concluded that this modality provides an effective and safe alternative method of pain palliation in patients with osteolytic metastases. A multicentre study involving 43 patients with painful osseous metastases was carried out by Goetz et al. [10] and showed again significant reduction of pain and decrease in the use of opioids, with only minor complications.

The proposed mechanisms by which RF ablation decreases pain may involve: pain transmission inhibition by destroying sensory nerve fibres in the periosteum and bone cortex; reduction of lesion volume with decreased stimulation of sensory nerve fibres; destruction of tumour cells that are producing nerve-stimulating cytokines (tumour necrosis factor-alpha [TNF-α], interleukins, etc.) and inhibition of osteoclast activity [11, 12].

In our patients, we observed a considerable reduction of pain and improvement of the quality of life, as measured by the BPI score.

A decrease in the use of analgesic medications was notable in our series and possibly greater than others reported in previous studies [9, 10].

The reduction of the procedural time is limited by both the time needed to achieve the optimal target temperature, and the size of the lesion, because more than one deployment is required in larger bone metastases. Although a few patients reported mild discomfort during the ablation, none of the sessions was forced to stop owing to considerable patient distress. There were none of the possible adverse effects, including infection, haemorrhage, neurological complications, skin burns, or the so-called post-ablation syndrome (low-grade fevers ≤100°F [37.8°C]), myalgias, and malaise for up to 1 week after the procedure). Pain reduction was fast and occurred within the first 24 h for some and during the first week in the majority of the patients. This appears to be a fairly well-tolerated procedure and the combination of conscious sedation and local anaesthesia is adequate for its needs.

In the spine, RF ablation may be contraindicated due to the close relationship with the spinal cord and nerve root. Vertebroplasty may be used for pain relief and stabilisation of osteolytic lesions. Pain relief occurs within hours or days (mean 24 h) of the procedure, sometimes after a transient worsening of pain [13]. Mechanical or thermal damage to the adjacent soft tissue from needle positioning or cement leakage are the potential complications.

In our study, there were no lesions threatening the stability of the spine.

There are two conflicting studies in the literature concerning the use of RF ablation in spinal metastases. One of them has demonstrated that the presence of cancellous or cortical bone between the lesion and the spinal canal can provide adequate safety for the procedure [14, 15]. Another study in an in vivo animal model, with the use of magnetic resonance imaging (MRI) and pathological evaluation, has demonstrated that the placement of the electrode against the posterior vertebral body wall resulted in damage of the spinal cord [16]. In most series, lesions within 1 cm of the spinal cord, lesions involving the posterior wall and lesions with cortical bone destruction with involvement of soft tissue were considered to represent contraindications to treatment [17]. All spinal metastases treated in our series involved the anterior part of the vertebral body.

The follow-up period for this study was 8 weeks, a period that we believed was sufficient to demonstrate that RFA provides effective palliation. There is, however, a need for randomised prospective studies, to evaluate the method and to compare it with other treatment modalities, such as radiation therapy. Continued follow-up is warranted to determine the long-term efficacy of this interventional approach.

In conclusion, imaged-guided RF ablation of painful bone metastases is promising. It appears to be effective, safe and well tolerated by patients.
